# Potatoes Consumption and Risk of Type 2 Diabetes: A Meta-analysis

**Published:** 2018-11

**Authors:** Yuanming ZHANG, Dingyun YOU, Nanjia LU, Donghui DUAN, Xiaoqi FENG, Thomas ASTELL-BURT, Pan ZHU, Liyuan HAN, Shiwei DUAN, Zuquan ZOU

**Affiliations:** 1. Dept. of Preventative Medicine, Zhejiang Provincial Key Laboratory of Pathophysiology, School of Medicine, Ningbo University, Ningbo, China; 2. Dept. of Science and Technology, Kunming Medical University, Kunming, China; 3. Population Wellbeing and Environment Research Lab (Power Lab), Faculty of Social Sciences, University of Wollongong, NSW, 2522, Australia; 4. Early Start Research Institute, Faculty of Social Sciences, University of Wollongong, Wollongong, NSW, 2522, Australia; 5. Menzies Center for Health Policy, School of Public Health, University of Sydney, Sydney, NSW, 2006, Australia

**Keywords:** Type 2 diabetes, Potatoes, French fries

## Abstract

**Background:**

Evidence of increased type 2 diabetes (T2D) risk associated with potatoes consumption is equivocal. We aimed to perform a meta-analyses on the association between potatoes consumption and T2D risk in prospective cohort studies.

**Methods:**

Studies published prior to 31 Aug 2016 were identified in PubMed, EMBASE, and Web of Science. Pooled relative risks (RR) and 95% confidence intervals (95%CI) based upon the highest vs. lowest category of potatoes consumption in each study were calculated in meta-analysis using random-effects models. Dose-response meta-analysis was fitted using generalized least squares regression in order to quantify the association between potatoes consumption and T2D risk.

**Results:**

The pooled RR comparing the highest vs. lowest category of potato consumption was 1.077 (95%CI: 1.005, 1.155). Dose-response meta-analysis revealed T2D risk increased 3.5% (RR=1.035, 95% CI: 1.004–1.067) for additional three serving per week serving of potato. The pooled RR comparing the highest vs. lowest category of French fries consumption was 1.362 (95%CI: 1.004, 1.850). Dose-response meta-analysis indicated T2D risk increased 18.7% (RR = 1.187, 95% CI: 1.067–1.321) for additional three serving per week of French fries.

**Conclusion:**

This meta-analysis support a significant positive association between high potatoes consumption and risk of T2D, especially the consumption of French fries.

## Introduction

Type 2 diabetes (T2D) is a global health concern. The number of people worldwide with T2D is expected to rise to 642 million by 2040 (1). Health expenditure due to T2D is predicted to exceed $490 billion by 2030 (2). WHO suggests T2D will become the seventh leading cause of death worldwide by 2030 (3).

Prevention of T2D is a major public health challenge (4). Although there is a genetic component, the pathogenesis of T2D also involves environmental factors that are potentially modifiable. Studies in nutritional epidemiology have highlighted the importance of dietary risk factors for T2D. Dose-response meta-analyses have reported increased T2D risk associated with ≥3 eggs consumed per week, low dairy intake and tea consumption of <3 cups/day (among women). More controversial is the evidence on T2D risk and potato consumption, with some studies, reported positive associations (5,6), whereas others suggested negative associations (7). Potatoes are the third most important food crop in the world after rice and wheat, more than 1 billion people worldwide eat potatoes, and global total crop production exceeds 374 million metric tons (8). Importantly, while potatoes belong to the vegetable group in US dietary guidelines (9), its consumption can influence glucose metabolism due to a large amount of starch easily absorbed (10). Potatoes have a high glycemic index (GI) and glucose load (GL) (11,12). Some studies evidenced significant association of high GI diet and GL with an increased risk of T2D (13–15). Furthermore, when the potatoes are heated, the starch becomes more digestible, which can result in raised blood sugar levels (16).

To provide clarification on this issue, we performed a dose-response meta-analyses of prospective cohort studies to quantify the association between potatoes consumption and T2D risk.

## Methods

### Literature search

This meta-analysis was conducted according to the guidelines of the meta-analysis of observational studies in epidemiology (17). We conducted a literature search of PubMed, Embase, Web of science up to 31 Aug 2016 (inclusive) for prospective cohort studies reporting associations between potatoes consumption and T2D. The language was restricted to English. In addition, we carefully reviewed the related articles, and hand searched reference lists. A complete search strategy was shown in ESI Text 1. To get additional data for this meta-analysis, we contacted authors for original data (18).

### Study selection

One author (YMZ) screened titles and abstracts, and two investigators (YMZ and NJL) reviewed full texts of potentially relevant articles and assessed study eligibility independently. Studies were included in this meta-analysis if they met the following inclusion criteria: 1) the study design was a prospective cohort study; 2) the exposure of interest was potatoes; 3) the endpoint of interest was T2D; 4) the relative risk (RR) or odds ratio (OR) or hazard ratio (HR) with 95% confidence interval (95%CI) for at least three categories of potato consumption were reported.

### Data extraction and quality assessment

Data extraction was completed by two independent authors (NJL and DHD), and the discrepancies in the extraction were resolved by a third party (DYY). The following data was extracted by using a template: last name of the first author; study name; year of publication; study location; length of follow-up; number of cases and participants; demographic characteristics (sex, age range); exposure (highest vs. lowest); the confounders adjusted in a multivariate analysis; and the adjusted RR, OR, or HR and 95%CIs in the corresponding exposure categories. In cases where the median or mean consumption for each category of potatoes consumption was not available, the midpoint of the upper and lower boundaries in each category was assigned as the average intake. If the lower or upper boundary for the lowest and highest category were not reported, the boundary had the same amplitude as the closest category (19). Potatoes included potato and French fries; potatoes referred to the boiled, baked and mashed potatoes. The Newcastle-Ottawa quality assessment scale which ranged from 0 (highest degree of bias) to 9 (lowest degree of bias), taking into account selection, comparability, and outcome assessment was used to assess the risk of bias in each study (20,21). We classified studies scoring 0–6 as low-quality and 7–9 as high-quality (20,21).

### Statistical analysis

For the dose-response meta-analysis, RRs and their 95%CIs from the highest vs. lowest category of potatoes consumption in each study were collected and pooled. HRs and ORs were treated as synonymous with RRs (22). Random effect models were used to calculate the pooled RRs (23). We tested heterogeneity of RRs across studies by using the Q statistic (significance level at *P*<0.10). The *I^2^* statistic was calculated to measure the variation across studies. We considered low, moderate, and high heterogeneity equivalent to *I^2^* values of 25%, 50%, and 75%, respectively (24,25). Finally, we conducted a sensitivity analysis to explore the influence of a single study on the combined risk estimate by omitting one study and analyzing the remainders in each turn (26).

Dose-response analysis was conducted based on the data for category of average potatoes consumption, the number of cases, person-years of follow-up, RRs and 95%CIs with generalized least squares regression (27,28). Potential publication bias was assessed using Begg’s funnel plots and Egger’s regression test (29). All analyses were performed using Stata v14.0 (StataCorp LP, College Station, TX). *P*<0.05 was considered statistically significant.

## Results

### Literature search

**Fig. 1:**
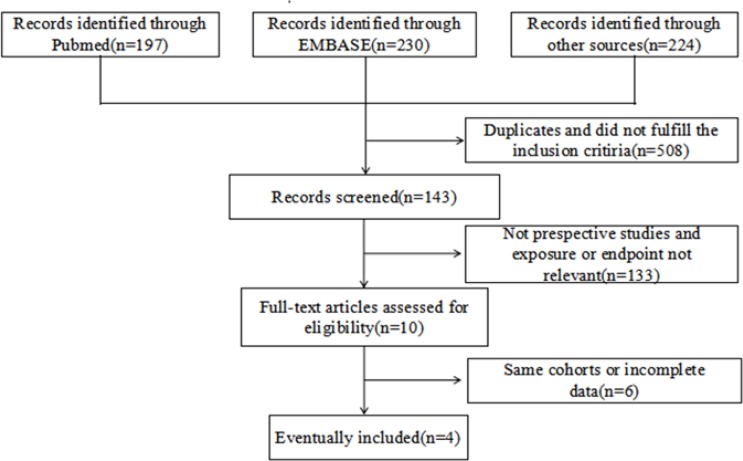
Flow chart of study selection

Overall, 651 articles were initially identified from PubMed, Embase and Web of science up to 31 Aug 2016 (inclusive). After exclusion of duplicates and studies that did not fulfill the inclusion criteria, we identified 143 potentially relevant studies. Two kinds of literature used the same cohort and we included only the most recently published (6,18). Finally, 6 eligible prospective cohort studies were included in our meta-analysis (6,7,18,30). Fig. 1 illustrates the flowchart of the literature selection.

### Study characteristics

The characteristics of the included studies were listed in Table 1. Of 6 prospective cohort studies, 4 prospective cohort studies were conducted in US (7,18), 1 in Finnish (6) and 1 in Australia (30).

**Table 1:** Characteristics of prospective cohort studies about potato consumption and T2D

***Reference No.***	***No. of participants/cases***	***Age range (yr)***	***Follow-up period (yr)***	***Exposure***	***RR(95% CI)***
Montonen et al (6)	4304/383	40–69	23	>283 v. <132 g/day	1.42 (1.02,1.98)
Liu et al. (7)	38018/1606	>45	8.8	0.93 v. 0.13 Servings per day	1.02 (0.86–1.22)
Muraki et al. HS (18)	70773/7436	41–59	26	&gE;5 v. <1 servings/week	1.03 (0.92, 1.15)
Muraki et al. (18)	87739/4621	30–43	20	&gE;5 v. <1 servings/week	1.17 (1.02, 1.35)
Muraki et al. (18)	40669/3305	42–65	24	&gE;5 v. <1 servings/week	1.06 (0.91, 1.23)
Hodge et al. (30)	31641/365	40–69	4	&gE;6.5 v. <2 servings/week	0.98 (0.70–1.37)

The participants were followed over a range of 4–26 yr. Of the 6 prospective cohort studies, the Nurses’ Health Study II had the largest number of participants (n=87739) (18), while the Finnish Mobile Clinic Health Examination Survey had the fewest (n=4304) (6). The adjusted confounding factors varied in different studies, and most of the risk estimates were adjusted for age, BMI and family history of diabetes. All of the included studies were considered high-quality studies according to the NOS (ESI Table 2).

**Table 2:** Characteristics of prospective cohort studies about French fries consumption and T2D

***Reference number***	***No. of participants/cases***	***Age range (yr)***	***Follow-up period (yr)***	***Exposure***	***RR(95% CI)***
(18)	70773/7436	41–59	26	≧5 v.<0.03 servings/week	1.45 (0.97, 2.16)
(18)	87739/4621	30–43	20	≧5 v.<0.03 servings/week	1.07 (0.85, 1.34)
(18)	40669/3305	42–65	24	≧5 v.<0.03 servings/week	1.68 (1.30, 2.17)

### The results of meta-analysis

**Fig. 2:**
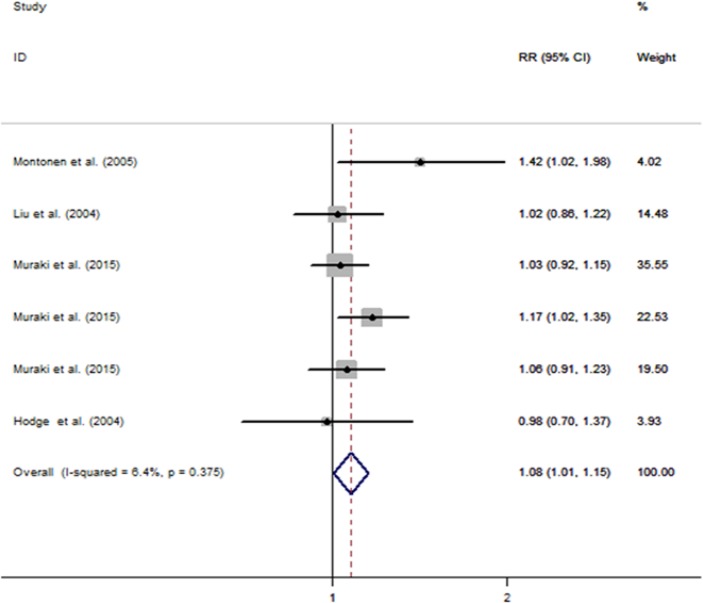
Forest plot of potato consumption and T2D risk

Fig. 2 showed the adjusted RRs for individual studies and pooled RRs for the highest vs. lowest category of potato consumption. In the six prospective cohort studies (6,7,18,30), we analyzed data from 273144 subjects (including 17716 T2D cases).

The pooled RR comparing the highest vs. lowest category of potato consumption was 1.077 (95%CI: 1.005, 1.155) in the random-effects model, with no heterogeneity among studies (*I^2^*=6.4%; *P*=0.375) (Fig. 2). The Begg’s funnel plot did not indicate any substantial asymmetry and the Egger’s regression test also did not reveal any strong evidence of publication bias (*P*=0.50) (Fig. 1).

**Fig. 3:**
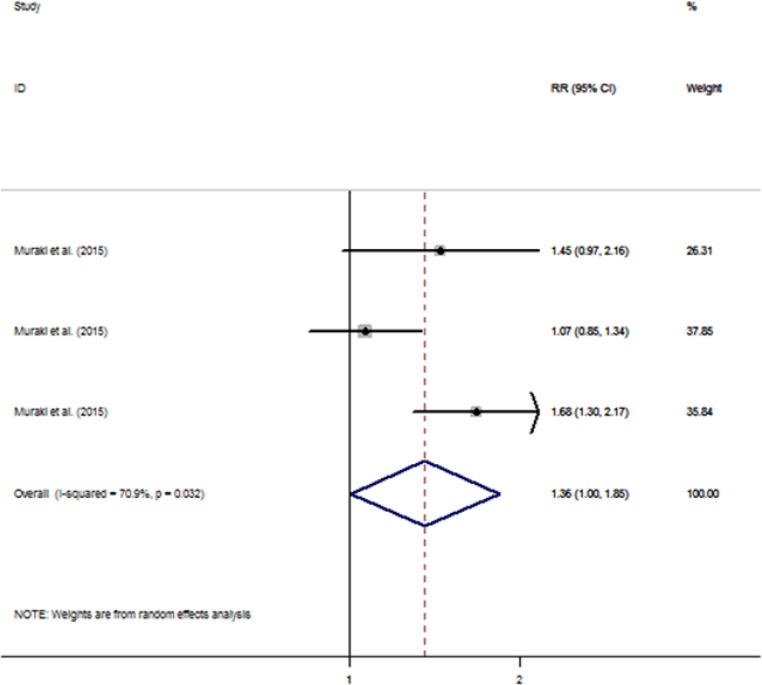
Forest plot of french fries consumption and T2D risk

Fig. 3 showed the adjusted RRs for individual study and pooled RR for the highest vs. lowest category of French fries consumption. Three prospective cohort studies were included (18). The pooled RR comparing the highest vs. lowest category of French fries consumption was 1.362 (95%CI: 1.004, 1.850) in the random-effects model, with heterogeneity observed among studies (*I^2^*=70.9%; *P*=0.03) (Fig. 3). The Begg’s funnel plot did not show any substantial asymmetry (Fig. 2). Egger’s regression test also indicated no evidence of publication bias (*P*=0.74).

### Sensitivity analysis

When we calculated potato consumption, the sensitivity analyses suggested the pooled RRs were not substantially modified by any single study, with a range from 1.050 (95% CI: 0.974, 1.133) to 1.103 (95% CI: 1.009, 1.205). Moreover, when we calculated French fries, the sensitivity analyses suggested our results were not substantially modified by any single study, with a range from 1.189 (95% CI: 0.895, 1.579) to 1.610 (95% CI: 1.297, 1.997).

## Dose-response analysis

**Fig. 4:**
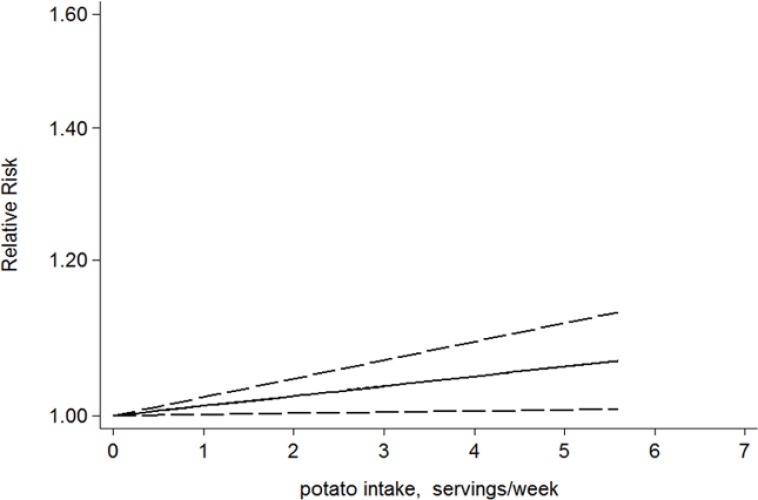
Linear dose-response relationship between potato consumption and T2D risk

When we analyzed potato consumption, this dose-response analysis involved in four studies. A two-stage random-effects dose-response model was performed to fit the linear dose-response relationship through generalized least squares regression, and a linear dose-response relationship was observed between potato consumption and the risk of T2D (*P*=0.026 for linearity). The dose-response analysis revealed that the risk of T2D was increased by 3.5%(RR=1.035, 95% CI: 1.004–1.067) for additional three serving per week of potato, the difference was statistically significant (*P*=0.026) (Fig. 4). When we analyzed French fries consumption, this dose-response analysis involved three studies.

**Fig. 5:**
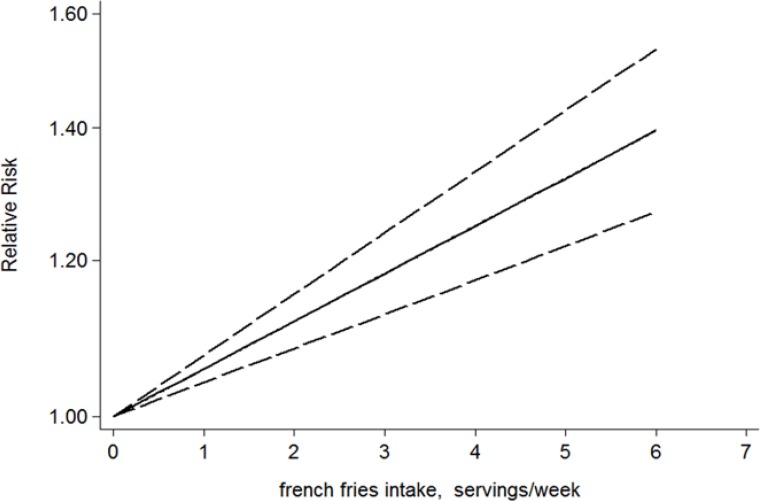
Linear dose-response relationship between french fries consumption and T2D risk

Similarly, a two-stage random-effects dose-response model was performed to fit the linear dose-response relationship through generalized least squares regression, and a linear dose-response relationship was observed between French fries consumption and the risk of T2D (*P*=0.0016 for linearity). The dose-response analysis revealed that the risk of T2D was increased by 18.7% (RR=1.187, 95% CI: 1.067–1.321) for additional three serving per week of French fries (Fig. 5). The difference was statistically significant (*P*=0.002).

## Discussion

Our dose-response meta-analyses of prospective cohort studies provide novel and important information on the association between potato or French fries consumption and T2D risk. Our meta-analysis demonstrated that T2D risk increased by 7.7% between the highest vs. lowest category of potato consumption. The dose-response meta-analysis found that the T2D risk was increased by 3.5% for additional three serving per week of potato. However, the combined results showed the risk of T2D was increased by 36.2% between the highest vs. lowest category of French fries consumption. The risk of T2D was increased by 18.7% for additional three serving per week of French fries. Thus, increased consumption of potato or French fries especially is associated with an elevated risk of T2D.

Compared to our study, a prospective cohort involving in 64,227 Chinese women without a history of T2D found that the risk was reduced by 28% for comparison between the highest vs. the lowest category of potato consumption (31). An increase in the consumption of potato during the 20-year follow-up was inversely related with 2-h glucose level (32). The other two studies showed that potato was positively associated with T2D (14,15). In a cross-sectional study involving 4774 Iranian adults, the frequency of potato consumption was associated with high fasting blood glucose level (33), T2D and low serum high-density lipoprotein level. High volumes of potato consumption significantly increased the risk of gestational diabetes mellitus (GDM)(34). All of those above evidence indicates the significant role of potato consumption on the progress of T2D.

Unlike other vegetables, potatoes have high GI and rich in starch, absorbed rapidly (10,35). High potatoes consumption may lead to a sharp rise in postprandial blood glucose concentrations, finally, result in β cell dysfunction or β cell exhaustion and the occurrence of T2D (36–38). In addition to the high GI, French fries consist of white potatoes and hydrogenated oils, which contains Trans-fat, associated with T2D risk (39). Furthermore, ingestion of high GL meal was associated with post-prandial hyperglycemia, which in turn contributed to endothelial dysfunction and inflammation (40). These mechanisms may explain how potato increase the T2D risk.

Our study has many strengths. Firstly, in order to reduce the potential for recall bias and reverse causation, our original literature search was restricted to prospective cohort studies. The latter is especially important since dietary changes usually encouraged post-T2D diagnosis by a family physician or general practitioner are likely to include cutting down on consumption of carbohydrates and ‘fast food’, including French fries (41). Secondly, the large number of participants in our meta-analyses increased the statistical power to identify reliable estimates. Thirdly, our dose-response meta-analysis revealed that the risk of T2D increased significantly with a three-unit increase in consumption of potatoes, especially when included French fries. Fourthly, no evidence of publication bias was found and the sensitivity analyses suggested the overall risk estimates were not substantially modified by any single study. Lastly, the results of all the studies included in our meta-analyses were adjusted for BMI and physical activity, known to be associated with T2D risk (42,43).

However, the limitations should also be noted. Firstly, currently, few studies focused on this topic. Since the studies included in our meta-analysis were mainly conducted in the United States, the generalizability of our findings may be limited. Secondly, the dietary guidelines for Americans regard potatoes as one of the vegetables (9) and United Kingdom’s national dietary guidelines believe potatoes belong to the starchy food group (44), but our findings contradict with the guidelines. Thirdly, adjustment for confounding was inconsistent between the studies included, which might result in some degree of distortion. Fourth, because potato and French fries are a common constituent part of a “western-style” diet and the complex lifestyle differences among different studies (45), it is difficult to disentangle the potential for interactions. Last, the GI of potatoes is not static, the GI is depended on what else is eaten and how the potato is cooked, but this is very difficult to obtain the accurate information of the above, besides, the commercial French fries (e.g. Macdonalds) are often dipped in dextrose, therefore the effect of French fries could actually be due to added ingredients. For future research, we make the following recommendations. Since all of the studies included in this analysis were observational, randomized controlled trials are encouraged. Meanwhile, the preferred methods of potatoes preparation and appropriate frequency of potato consumption are future concerns. Frying, microwaving and baking could decrease the postprandial glycemic response significantly in comparison with boiling or mashing, and suggested an effective way to lower the GI of potato through cooling or co-digestion with protein, lipid and vinegar (46).

## Conclusion

The meta-analyses of prospective cohort studies provided evidence of a significant positive association between high potatoes consumption and risk of T2D, especially the consumption of French fries. This supports previous calls for substitution of baked, boiled, or mashed potatoes with whole grains or other healthier alternatives to potentially lower T2D risk.

## Ethical considerations

Ethical issues (Including plagiarism, informed consent, misconduct, data fabrication and/or falsification, double publication and/or submission, redundancy, etc.) have been completely observed by the authors.
